# Patient Surveillance Adherence After Treatment for Endometrial Cancer

**DOI:** 10.1097/og9.0000000000000010

**Published:** 2024-05-30

**Authors:** Naixin Zhang, Lila Marshall, Sarah Thappa, Alexandra Morell, Alexandra Samborski, Richard Moore, MaryAnn Wilbur

**Affiliations:** Division of Gynecologic Oncology, Department of Obstetrics and Gynecology, and the Department of Obstetrics and Gynecology, University of Rochester Medical Center, Rochester, New York.

## Abstract

**BACKGROUND:**

To assess factors that may affect patients' adherence to recommended surveillance after treatment for endometrial cancer.

**METHODS:**

We performed a retrospective study of all patients undergoing primary treatment for endometrial cancer from January 1, 2010, to December 30, 2017, at a tertiary referral center. Patients were included for analysis if there was sufficient information in the electronic medical records. Patient demographic information, cancer characteristics, and surveillance adherence data were collected. The median inflation-adjusted income was calculated from the patients' ZIP codes and U.S. Census data. The primary outcome assessed was *adherence to surveillance*, defined as two or more visits per 12 months in individuals at low risk and four or more visits per 12 months in individuals at high risk, consistent with recommended guidelines.

**RESULTS:**

During the study period, 870 patients were included for analysis. The mean age at diagnosis and body mass index (BMI) of the entire cohort were 63.4 years and 35.8, respectively. Overall, 761 patients (87.5%) were adherent to the recommended surveillance guidelines. Patients who were not adherent to recommended surveillance lived significantly farther from the cancer center (39.2 miles vs 20.7 miles, *P*=.026) and had a significantly lower median inflation-adjusted income ($74,015 vs $80,435, *P*=.027).

**CONCLUSION:**

Increased distance to a tertiary care center and lower median income were significantly associated with decreased adherence to recommended surveillance guidelines. This is consistent with current literature on increased distance traveled as a proxy for worse survival in gynecologic malignancies, as well as worse endometrial cancer outcomes in patients of lower socioeconomic status. This highlights an area of disparity that needs improvement and warrants further investigation.

## Abstract 2

Increased distance to a tertiary care center and lower income were associated with decreased adherence to surveillance guidelines after primary treatment of endometrial cancer.

Endometrial cancer is the most common gynecologic malignancy in the United States, with an estimated 66,000 new cases and 12,500 deaths in 2022.^1^ Primary treatment depends on several factors, namely stage at diagnosis and histopathologic variables. Cancer confined to the uterus with no high-risk features typically requires only surgical excision. However, adjuvant treatment with systemic therapy or radiation therapy may be warranted in cases with high-risk features.^2–6^ Regardless of treatment modality, treatment adherence is a predictor of improved survival, and posttreatment surveillance is recommended for all patients to monitor for recurrence.^7^

Many different tests and modalities are used for surveillance, with physical examination being the most common and effective.^8–10^ The Society of Gynecologic Oncology (SGO) originally published recommended guidelines for posttreatment surveillance for endometrial cancer in 2011, recommending physical examination every 3–6 months for 2 years and then every 6 months or annually thereafter.^11,12^ These guidelines have since been adopted by the National Comprehensive Cancer Network (NCCN) and have helped set the standard for diagnosing recurrence after treatment and identify ways to decrease health care costs without compromising quality of care.^13,14^ Ideally, every patient with a history of endometrial cancer would have the ability and resources to adhere to the recommended guidelines for both treatment and surveillance. Several studies have evaluated nonadherence to recommended treatment guidelines for endometrial cancer and outcomes related to surveillance adherence; however, there is a paucity of data on the adherence to recommended surveillance guidelines after treatment.^15–18^ We sought to evaluate factors that may be associated with decreased adherence to surveillance guidelines.

## METHODS

This University of Rochester Medical Center IRB–approved study was performed with available retrospective available data, and consent requirements were waived. We performed a retrospective study of all patients with a diagnosis of endometrial cancer (International Classification of Diseases, Tenth Revision code 56.1) attached to their electronic medical record from January 1, 2010, to December 30, 2017, at a tertiary referral center. Patients were excluded from analysis if they had the incorrect primary cancer type identified or if there were insufficient data in the electronic medical records regarding their complete treatment history and surveillance course. Patient demographic information, cancer characteristics, and surveillance adherence data were collected. Race or ethnicity was obtained by chart review and included in the study because of their potential role in health care disparities.^19–21^ Cancer staging was performed surgically through laparotomy or minimally invasive surgery. The primary outcome assessed was adherence to surveillance. *Adherence to surveillance* was defined as two or more visits per 12 months in individuals at low risk and four or more visits per 12 months in those at high risk, consistent with the NCCN and SGO recommended guidelines.^11,12^ The median, inflation-adjusted income was calculated from the patients' ZIP codes and U.S. Census data.^22^ The distance from a patient's home to the hospital was measured in miles with Google Maps.^23^

All statistical analyses were performed in SPSS 27. We used χ^2^, Mann–Whitney *U*, and *t* test analyses to evaluate for significant differences between the two cohorts as appropriate. The Fisher exact test was used as appropriate. All *P* values were two-sided, with statistical significance defined as *P*<.05.

## RESULTS

A total of 1,046 patients with endometrial cancer were identified. Thirty-three patients were excluded for having the incorrect primary cancer type or diagnosis (13 cervical, two vulvar, three ovarian, one colorectal, four sarcoma, four benign), and 143 were excluded for having insufficient information in the electronic medical records. As a result, a total of 870 patients were included for analysis. The mean age at diagnosis and body mass index (BMI, calculated as weight in kilograms divided by height in meters squared) for the entire cohort were 63.4 years and 35.8, respectively. Apart from BMI (35.3 vs 39.0, *P*<.005), there were no other significant demographic or comorbidity differences between patients who were and those who were not adherent to surveillance recommendations. Patient characteristics are shown in Table 1. Overall, 761 patients (87.5%) were adherent to the recommended posttreatment surveillance guidelines. Patients who were not adherent to the recommended surveillance lived significantly farther from the cancer center (39.2 miles vs 20.7 miles, *P*=.026) and had a significantly lower median inflation-adjusted income ($74,015 vs $80,435, *P*=.027). Fifty-five patients (6.3%) identified as Hispanic or Black. Patients who identified as Hispanic or Black had an adherence rate of 80.0% compared with 88.0% for patients who identified as White or another race or ethnicity, although this discrepancy was not statistically significant (*P*=.08). There was no significant difference in posttreatment surveillance adherence rate between patients with public insurance (Medicare, Medicaid) and those with private insurance (*P*=.684). There were no significant differences between the two cohorts in terms of cancer histology. The adherent cohort was noted to have a higher percentage of patients with stage II cancer (5.7% vs 0.9%, *P*=.033) and a lower percentage of patients with unstaged cancer (1.2% vs 6.4%, *P*<.005). Cancer characteristics are shown in Table 2.

**Table 1.** Patient Characteristics

Characteristic	Adherent (n=761)	Not Adherent (n=109)	*P*
Age (y)	63.6±10.5	62.2±11.0	.196
Race and ethnicity			
Black	33 (4.3)	9 (8.3)	.068
Hispanic	11 (1.4)	2 (1.8)	.672
White (non-Hispanic)	706 (92.8)	98 (89.9)	.285
None of the above	11 (1.4)	0 (0.0)	.377
BMI (kg/m^2^)	35.3±9.3	39.0±11.4	**<.005**
Postmenopausal	658 (86.5)	94 (86.2)	.986
Median income ($)	80,435	74,015	**.027**
Median distance (miles)	20.7	39.2	**.026**
Medical history			
Hypertension	466 (61.2)	71 (65.1)	.706
Diabetes	249 (32.7)	47 (43.1)	.145
COPD	46 (6.0)	11 (10.1)	.144
CKD (mild to moderate)	86 (11.3)	13 (11.9)	.864
History of MI or CVA	56 (7.4)	11 (10.1)	.360
Public insurance	490 (64.4)	68 (62.4)	.684

BMI, body mass index; COPD, chronic obstructive pulmonary disease; CKD, chronic kidney disease; MI, myocardial infarction; CVA, cerebrovascular accident.

Data are mean±SD or n (%) unless otherwise specified.

Bold indicates statistical significance.

**Table 2.** Cancer Characteristics

Characteristic	Adherent (n=761)	Not Adherent (n=109)	*P*
Cancer stage			
I	585 (76.9)	89 (81.7)	.262
II	43 (5.7)	1 (0.9)	**.033**
III	92 (12.1)	11 (10.1)	.546
IV	32 (4.2)	1 (0.9)	.109
Not staged	9 (1.2)	7 (6.4)	**<.005**
Cancer histology			
Endometrioid	599 (78.7)	99 (90.8)	.337
Papillary serous	84 (11.0)	6 (5.5)	.110
Carcinosarcoma	17 (2.2)	0 (0)	.151
Clear cell	6 (0.8)	1 (0.9)	.889
Mixed or other	37 (4.9)	3 (2.8)	.464

Data are n (%) unless otherwise specified.

Bold indicates statistical significance.

## DISCUSSION

Overall, our patient population had an 87.5% adherence rate to the recommended posttreatment surveillance for endometrial cancer. Distance from the cancer center and lower median inflation-adjusted income were associated with decreased patient adherence to recommended surveillance guidelines after treatment for endometrial cancer. Race and ethnicity were not found to be significant factors regarding surveillance.

The SGO initially published recommended guidelines for posttreatment surveillance for endometrial cancer in 2011, recommending physical examination every 3–6 months for 2 years and then every 6 months or annually thereafter.^11,12^ These guidelines have since been adopted by the NCCN and have helped identify ways to decrease health care costs without compromising quality of care, namely avoiding unnecessary testing such as routine vaginal Pap tests, CA 125, and imaging.^13,14^ Schwartz et al^24^ investigated the effects of these guidelines on our health care system and noted a significant decrease in unnecessary testing with associated decreased health care costs without compromising patient outcomes. Furthermore, the less intensive surveillance protocols (physical examination only while omitting routine serologic, cytologic, or imaging tests) for endometrial cancer may have no difference in overall survival compared with more intensive protocols.^25^ This reinforces that the guidelines are effective and that counseling patients on the importance of surveillance should be a priority. Our study showed that our patient population had an 87.5% adherence rate to the recommended posttreatment surveillance for endometrial cancer. This is one of the first studies specifically examining factors affecting patient adherence to recommended surveillance guidelines for endometrial cancer.^15–18^ Understanding the patterns of patient adherence is important because adequate surveillance is associated with high recurrence detection rates, and early detection is associated with better outcomes.^11^ If specific patient populations are found to have decreased adherence rates, further study should be initiated to explore the reasons for disparities in the delivery of care.

We noted statistically significant lower surveillance adherence rates in patients who lived farther from the medical center or had a lower median income. These findings were consistent with factors associated with nonadherence to recommended treatment in endometrial cancer and poorer outcomes in other gynecologic malignancies.^16,26,27^ These factors likely affect patients' ability to comply with the frequent visits recommended for surveillance. Farther distances to travel, transportation limitations, and work schedule conflicts could hinder patients' attendance at surveillance visits. Of note, our study did not assess whether patients attended surveillance visits through outside clinicians because this is not typical in our practice. Because our median distance difference between the two groups was only 18 miles, further study may be warranted to evaluate potential surveillance through outside clinicians in tertiary care centers with larger catchment areas. In addition, we found that the nonadherent cohort had a significantly higher BMI. This is likely the result of confounding because higher BMI is associated with lower socioeconomic status.^28^

Our study did not find a significant difference in adherence to surveillance in patients who identified as Black or Hispanic. We suspect that this study is underpowered to meet statistical significance for race or ethnicity because 6.3% of the study population identified as Hispanic or Black. This is important to note given the known racial and ethnic disparities characteristic of not only endometrial cancer but health care as a whole.^16,19–21^ Further study is indicated in a more diverse patient cohort to better characterize the association of race with adherence rates. There was also no statistically significant difference in patients' adherence to surveillance based on histology, so those with less aggressive cancer types were just as likely as those with more aggressive cancer types to follow up according to recommendations. Patients with stage II disease were more likely to adhere to surveillance guidelines, whereas those with unstaged disease were less likely. We hypothesize that there are psychosocial confounders associated with both factors; for example, patients with decreased access to health care (namely because of income or distance to a hospital) were less likely to be able to seek care in a timely fashion; thus, the cancer may have spread to a point where surgical staging is not feasible.^29,30^ Fortunately, the rate of adherence to surveillance in this study was high because it is particularly important that patients with a high risk of recurrence based on histology or stage understand their risk and follow up appropriately.

The strength of this study is that it is one of the first studies aimed specifically at identifying factors affecting posttreatment surveillance adherence. This is an underexplored area in the field and can help identify causes of health care disparities. Limitations of this study include the retrospective nature and the lack of racial and ethnic diversity of the patient population. Our patient population overwhelmingly identified as non-Hispanic White; thus, we may not have had the power to detect race- or ethnicity-associated factors for surveillance adherence, and our findings may not be as generalizable. In addition, adjuvant treatment information was not collected because the primary purpose was surveillance adherence after upfront treatment. Different adjuvant treatments could potentially affect surveillance adherence and warrant further study.

This study shows a high rate of adherence to surveillance visits for patients with endometrial cancer, although there is a paucity of data for comparison. Distance from the cancer center and lower median inflation-adjusted income are factors associated with decreased patient adherence to recommended surveillance guidelines after treatment for endometrial cancer. This highlights an area of disparity that needs improvement and warrants further investigation.
